# LMDmapper: an open-source desktop tool for spatial mapping of laser microdissection samples

**DOI:** 10.12688/openreseurope.24543.1

**Published:** 2026-07-24

**Authors:** Antton Alberdi, Jaime Ramirez, Nanna Gaun, Zoé Horisberger, Bryan Wang, Urvish Trivedi, Amalia Bogri

**Affiliations:** 1 Globe Institute, University of Copenhagen, Copenhagen, Capital Region of Denmark, Denmark; 2 Department of Biology, University of Copenhagen, Copenhagen, Capital Region of Denmark, Denmark

**Keywords:** Imaging, Laser capture microdissection, Micro-scale, Spatial metagenomics, Specimen

## Abstract

Laser microdissection (LMD) enables researchers to isolate targeted microsamples from microscopy slide specimens for downstream molecular analyses. While traditionally employed for isolating eukaryotic cells from complex tissues, LMD is starting to be used for micro-scale spatial microbiome analyses, which require the precise location of the microsamples to be tracked for downstream spatial analyses. To address this need, we present LMDmapper, an open-source desktop application that allows designing, tracking and logging micron-scale spatial microsample data and metadata from LMD sessions. The software parses Leica Database LIF image files (containing stage coordinates), imports laser microdissection CSV exports (containing image pixel coordinates), transforms and maps image pixel coordinates into stage coordinates, and presents the resulting cut points together with user-defined plate layouts and collection metadata. LMDmapper supports a variety of microdissection designs, including multiple slides, specimens, collection plates and plate layouts. The application is implemented in TypeScript using Electron, React, Vite, and fast-xml-parser. LMDmapper outputs include a metadata CSV linking microsample identifiers to plate positions, collection information, image labels, pixel coordinates and stage coordinates, as well as the possibility to create overview images of the specimens, and automatically calculating distances between cutting points and regions of interest. With these capabilities, LMDmapper is intended as a practical bridge between microscope-side laser microdissection records and downstream spatial omics sample tracking.

## Plain Language summary

Laser microdissection is a technique that lets researchers cut very small regions from a thin specimen section, such as selected cells or gut content, and collect them for DNA, RNA, protein, or metabolite measurements. Often, the scientific value of these measurements depends on knowing exactly where every collected sample came from. This can become difficult because the information is spread across microscope image files, exported tables, plate maps, collection notes, and later sample names. LMDmapper is a desktop application that helps researchers keep these pieces connected. A user can define a project layout, import microscopy imaging files, calculate the stage coordinates of each cut, inspect missing or ambiguous image links, manually correct coordinates when needed, and export a clean metadata table for downstream analysis. The tool also lets users align overview images and export cropped overview regions with the positions of collected samples. The software is open source and can be run as a packaged desktop application on macOS, Windows, and Linux. It is designed for laboratory workflows where careful tracking of microscopic sample position, plate well, image evidence, and microsample identity is needed after laser microdissection.

## Introduction

Laser capture microdissection, also called laser microdissection (LMD), is used to isolate defined small regions of interest from heterogeneous samples under direct microscopic visualisation. ^1^ This physical isolation preserves spatial context while enabling downstream molecular assays such as genomic, transcriptomic, proteomic, and metabolomic analyses. ^1^ ^,^ ^2^ The method is particularly useful in cases when bulk homogenisation would mix molecular signals from adjacent compartments. ^2^

Modern laser microdissection workflows are increasingly connected to spatial biology and image-analysis pipelines. Commercial laser microdissection software provides tools for selecting, dissecting, navigating, and documenting cuts, including sample overviews, collector tracking, and shape-location records. Other open-source tools address complementary parts of the workflow, such as transferring regions of interest between serial sections, ^3^ or integrating image-analysis algorithms with laser dissection platforms. ^4^

Despite these advances, post-dissection curation remains difficult in workflows where microscope image files, cut-coordinate exports, collector positions, plate configurations, and downstream sample identifiers are stored as separate records. In Leica LMD workflows, LIF (Leica Image File Format; *.lif ) files contain stage coordinates (μm), image data, and acquisition metadata, while CSV exports contain complementary information such as cut shapes, image labels, and pixel coordinates (px). The lack of an accessible tool for reconciling these records makes it difficult to build spatially aware LMD sample libraries with traceable Cartesian coordinates and supporting image evidence.

LMDmapper addresses this post-dissection curation need. The tool is not intended to drive the microscope or generate cutting instructions. Instead, it focuses on mapping and validating the relationship between Leica LIF images, laser microdissection CSV exports, specimen and plate layouts, collector positions, cut-point coordinates, collection metadata, and overview images. The aim is to provide an auditable bridge between laser microdissection acquisition records and the structured metadata required for downstream molecular analysis.

## Methods

### Implementation

LMDmapper is implemented as a cross-platform desktop application using Electron, ^5^ React, ^6^ Vite, ^7^ and fast-xml-parser. ^8^ The core parsing procedure of the software, where output files of the Leica instrument are reconciled with the metadata, is written in TypeScript ^9^ and operates locally on the user’s files. The software addresses a major limitation of the LIF files with regard to the absolute spatial coordinates of the cutting position through a two-step process.

First, LMDmapper reads the `LMSDataContainerHeader` XML section of the LIF files using UTF-8 or UTF-16LE detection, and parses metadata from `Element` nodes. For each microscopy image capture, LMDmapper extracts the element name, memory block identifier, memory size, dimensions, channel count, bit resolution, stage position, collector holder position, acquisition timestamp, and laser settings. The parser currently marks an element as display-supported when it is 2D RGB, 8-bit, three-channel, interleaved image data and the recorded memory size matches width times height times three bytes. The parser caches file-level metadata and resolves image data by scanning the binary section for memory block tokens in ASCII or UTF-16LE. It can load full images or generate reduced-size thumbnails. Stage positions (μm) are read from Leica metadata fields, normalised internally, and used as the centre point of an image element in the coordinate map.

Second, the complementary CSV parser reads laser microdissection export fields. This file includes the pixel coordinates (px) indicating the exact location of the laser cutting points within the image element recorded in the previous step, which is essential to calculate the absolute position of the microsample cutting point. Finally, LMDmapper combines the stage coordinates with the pixel coordinates to yield the absolute Cartesian coordinates of all cutting points.

### Operation

**Figure 1.**
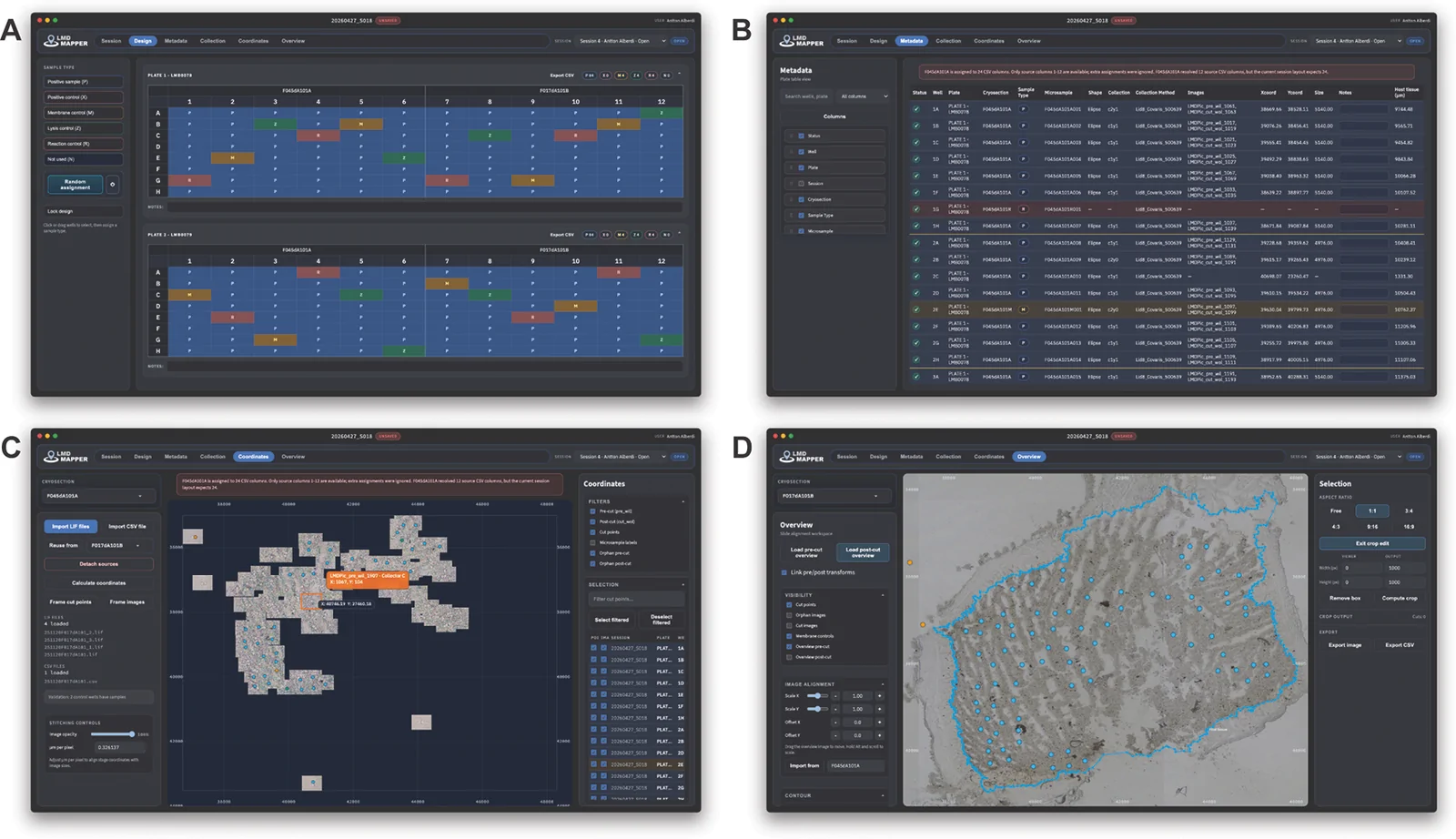
Screenshots of four workflow tabs. **A)** Design page showing the randomly assigned locations of different types of controls and regular samples across two plates. **B)** Metadata page containing sample details in tabular format. **C)** Coordinates page after loading the LIF and CSV files. Cutting points are indicated by blue markers, with the cursor position and details of the hovered image displayed in tooltips. A warning sign indicating unexpected sample information is shown above the canvas. **D)** Overview page showing cutting locations overlaid on a full-slide image. Blue dots represent regular samples, while orange dots represent membrane controls. A manually generated blue contour delineates the host tissue and is used to automatically calculate distances to the closest reference.

LMDmapper can be used as a packaged desktop application on Windows (10+), macOS (Monterey 12+), and Linux (Ubuntu 18.04+, Fedora 32+, Debian 10+) operating systems. The software contains six workflow tabs that are used before (Project and Design), during (Collection) and after (Metadata, Coordinates, and Overview) the laser microdissection session ( Fig. 1). The expected user workflow is:

Before the LMD session:

1. **Start a session:** Define the session users to log changes.
2. **Configure the project**: Declare session name, cryosection or specimen identifiers, stage positions, number of plates, and plate-to-cryosection assignments. LMDmapper supports multiple sampling designs.
3. **Define the plate design**: For each of the microsample collecting plates, define the location of positive samples, controls, disabled wells, and microsample numbering ( Fig. 1a).

During the LMD session:

4. **Log microsample collection**: For each microsample cutting, declare the number of attempts for generating the sample and the number of microsamples visually checked in the collector.

After the LMD session:

5. **Import files**: Load Leica LIF files and laser microdissection CSV files for each cryosection to calculate laser microdissection locations and visualise them in a two-dimensional space ( Fig. 1b).
6. **Inspect the coordinate map**: Check image footprints, fix cut points, and detect missing or suspicious assignments ( Fig. 1c).
7. **Enter collection metadata**: Enrich the metadata of each session with plate notes, collection method, date, time, temperature, humidity, and free-text notes.
8. **Export metadata**: Choose visible and exported columns, and export a metadata CSV.
9. **Export image overview**: Align overview images, draw contours, compute crop cut positions, and export overview crops as PNG and CSV files ( Fig. 1d).
10. **Save the session**: As a `*.lmd` file for a single session or combine multiple saved sessions into a `.mlmd` workspace.

## Use cases

### Curating a single laser microdissection session

A common LMDmapper use case is a session with one or two 96-well plates and one or more cryosections. The user first records the session name, cryosection identifiers, stage positions, and plate layout. Wells are classified as containing samples, controls, or not used. LMDmapper then generates microsample codes that remain tied to the plate and cryosection design.

The user imports the LIF files and CSV exports for each cryosection. LMDmapper parses the image metadata, reconstructs source columns from the CSV rows, routes the source columns onto the configured plate segments, and computes stage coordinates for wells with linked image names and pixel coordinates. The Metadata tab then provides one row per plate well, including plate, well, cryosection, sample type, microsample code, collection label, image labels, pixel coordinates, stage coordinates, size, status, notes, and optional distances to user-defined contours. Each row links the downstream microsample identifier to its tissue-derived coordinate evidence and collection context. For wells without valid coordinate evidence, LMDmapper marks the row as pending, warning, or bad depending on sample type and available information.

### Resolving missing image links and orphan images

Laser microdissection exports may contain incomplete image labels or may be missing images. LMDmapper handles these cases by distinguishing linked image records from orphan images. Orphan images are image elements present in the `*.lif` file but not assigned to a resolved metadata row.

In the Coordinates view, the user can filter orphan pre-cut and post-cut images, group them by collector row, inspect hover metadata, and assign an orphan image to a missing plate position. LMDmapper can also preserve original CSV pixel coordinates for a well even when its image link is removed, allowing the predicted cut marker to be displayed during reassignment. If no image evidence is available, the user can enter manual stage coordinates and link them to a metadata row. Manual coordinates and manual image assignments remain in warning status until explicitly confirmed, preserving an audit trail for downstream users.

The expected output is a corrected metadata table in which formerly missing or ambiguous wells have explicit manual-assignment status, image labels where available, resolved coordinates, and user notes describing the correction.

### Exporting overview crops and spatial annotations

For reporting, quality control, or downstream spatial analysis, users may need an overview image crop that contains a subset of collected cut points. LMDmapper allows the user to import pre-cut and post-cut overview images, align them to the stage coordinate system, and show cut points, image footprints, orphan images, and control samples. The user can draw a selection rectangle, set an export size, compute which cut points fall within the crop, and export both the rendered crop image PNG and a CSV table of crop-local pixel coordinates. Users can also draw contours on the overview image, such as tissue boundaries or regions of interest. LMDmapper calculates the shortest distance from each cut point to each visible contour and exposes those distances as optional metadata columns.

## Discussion

LMDmapper addresses a practical reproducibility problem in laser microdissection workflows: Maintaining a transparent link between image evidence, spatial coordinates, plate positions, collection records, and downstream sample identifiers. By keeping the session state in structured local files and exporting plain CSV outputs, the tool is designed to fit into downstream omics and laboratory information-management workflows without requiring a server or proprietary database export.

The application is most useful when a dissection workflow has enough complexity that manual spreadsheet curation becomes error-prone. Examples include split plates, multiple cryosections, positive and negative controls, shared CSV sources, re-cuts, missing image labels, manually corrected coordinates, and multiple saved sessions that need to be compared. The explicit warning states for inferred or manual assignments are intended to reduce hidden curation bias by making uncertain mappings visible at export time.

## Ethics and consent

Ethical approval and consent were not required.

## Data Availability

Source code available from:
-

github.com/anttonalberdi/lmdmapper

- Archived software available from: 10.5281/zenodo.20827562
- License: MIT License.
