# LMDmapper: an open-source desktop tool for spatial mapping of laser microdissection samples

**DOI:** 10.12688/openreseurope.24543.2

**Published:** 2026-09-08

**Authors:** Antton Alberdi, Jaime Ramirez, Nanna Gaun, Zoé Horisberger, Bryan Wang, Urvish Trivedi, Amalia Bogri

**Affiliations:** 1 Globe Institute, University of Copenhagen, Copenhagen, Capital Region of Denmark, Denmark; 2 Department of Biology, University of Copenhagen, Copenhagen, Capital Region of Denmark, Denmark

**Keywords:** Imaging, Laser capture microdissection, Micro-scale, Spatial metagenomics, Specimen

## Abstract

Laser microdissection (LMD) enables researchers to isolate targeted microsamples from microscopy slide specimens for downstream molecular analyses. While traditionally employed for isolating eukaryotic cells from complex tissues, LMD is starting to be used for micro-scale spatial microbiome analyses, which require the precise location of the microsamples to be tracked for downstream spatial analyses. To address this need, we present LMDmapper, an open-source desktop application that allows designing, tracking and logging micron-scale spatial microsample data and metadata from LMD sessions. The software parses Leica Database LIF image files (containing stage coordinates), imports laser microdissection CSV exports (containing image pixel coordinates), transforms and maps image pixel coordinates into stage coordinates, and presents the resulting cut points together with user-defined plate layouts and collection metadata. LMDmapper supports a variety of microdissection designs, including multiple slides, specimens, collection plates and plate layouts. The application is implemented in TypeScript using Electron, React, Vite, and fast-xml-parser. LMDmapper outputs include a metadata CSV linking microsample identifiers to plate positions, collection information, image labels, pixel coordinates and stage coordinates, as well as the possibility to create overview images of the specimens, and automatically calculating distances between cutting points and regions of interest. With these capabilities, LMDmapper is intended as a practical bridge between microscope-side laser microdissection records and downstream spatial omics sample tracking.

## Plain Language summary

Laser microdissection is a technique that lets researchers cut very small regions from a thin specimen section, such as selected cells or gut content, and collect them for DNA, RNA, protein, or metabolite measurements. Often, the scientific value of these measurements depends on knowing exactly where every collected sample came from. This can become difficult because the information is spread across microscope image files, exported tables, plate maps, collection notes, and later sample names. LMDmapper is a desktop application that helps researchers keep these pieces connected. A user can define a project layout, import microscopy imaging files, calculate the stage coordinates of each cut, inspect missing or ambiguous image links, manually correct coordinates when needed, and export a clean metadata table for downstream analysis. The tool also lets users align overview images and export cropped overview regions with the positions of collected samples. The software is open source and can be run as a packaged desktop application on macOS, Windows, and Linux. It is designed for laboratory workflows where careful tracking of microscopic sample position, plate well, image evidence, and microsample identity is needed after laser microdissection.

## Introduction

Laser capture microdissection, also called laser microdissection (LMD), is used to isolate defined small regions of interest from heterogeneous samples under direct microscopic visualisation. ^1^ This physical isolation preserves spatial context while enabling downstream molecular assays such as genomic, transcriptomic, proteomic, and metabolomic analyses. ^1^ ^,^ ^2^ The method is particularly useful in cases when bulk homogenisation would mix molecular signals from adjacent compartments. ^2^

Modern laser microdissection workflows are increasingly connected to spatial biology and image-analysis pipelines. Commercial laser microdissection software provides tools for selecting, dissecting, navigating, and documenting cuts, including sample overviews, collector tracking, and shape-location records. Other open-source tools address complementary parts of the workflow, such as transferring regions of interest between serial sections, ^3^ or integrating image-analysis algorithms with laser dissection platforms. ^4^

Despite these advances, post-dissection curation remains difficult in workflows where microscope image files, cut-coordinate exports, collector positions, plate configurations, and downstream sample identifiers are stored as separate records. In Leica LMD workflows, LIF (Leica Image File Format; *.lif ) files contain stage coordinates (μm), image data, and acquisition metadata, while CSV exports contain complementary information such as cut shapes, image labels, and pixel coordinates (px). The lack of an accessible tool for reconciling these records makes it difficult to build spatially aware LMD sample libraries with traceable Cartesian coordinates and supporting image evidence.

LMDmapper addresses this post-dissection curation need. The tool is not intended to drive the microscope or generate cutting instructions. Instead, it focuses on mapping and validating the relationship between Leica LIF images, laser microdissection CSV exports, specimen and plate layouts, collector positions, cut-point coordinates, collection metadata, and overview images. The aim is to provide an auditable bridge between laser microdissection acquisition records and the structured metadata required for downstream molecular analysis.

## Methods

### Implementation

LMDmapper is implemented as a cross-platform desktop application using Electron, ^5^ React, ^6^ Vite, ^7^ and fast-xml-parser. ^8^ The core parsing procedure of the software, where output files of the Leica instrument are reconciled with the metadata, is written in TypeScript ^9^ and operates locally on the user’s files. The software addresses a major limitation of the LIF files with regard to the absolute spatial coordinates of the cutting position through a two-step process.

First, LMDmapper reads the ‘LMSDataContainerHeader’ XML section of the LIF files using UTF-8 or UTF-16LE detection, and parses metadata from ‘Element’ nodes. For each microscopy image capture, LMDmapper extracts the element name, memory block identifier, memory size, dimensions, channel count, bit resolution, stage position, collector holder position, acquisition timestamp, and laser settings. The parser currently marks an element as display-supported when it is 2D RGB, 8-bit, three-channel, interleaved image data and the recorded memory size matches width times height times three bytes. The parser caches file-level metadata and resolves image data by scanning the binary section for memory block tokens in ASCII or UTF-16LE. It can load full images or generate reduced-size thumbnails. Stage positions (μm) are read from Leica metadata fields, normalised internally, and used as the centre point of an image element in the coordinate map.

Second, the complementary CSV parser reads laser microdissection export fields. This file includes the pixel coordinates (px) indicating the exact location of the laser cutting points within the image element recorded in the previous step, which is essential to calculate the absolute position of the microsample cutting point. Finally, LMDmapper combines the stage coordinates with the pixel coordinates to yield the absolute Cartesian coordinates of all cutting points.

All outputs are written in open, documented formats so that curated records can leave the application without a converter. Tabular outputs — the metadata table, the plate configuration, and the overview crop coordinates — are exported as CSV, and session state is retained as plain JSON. Spatial annotations are exported as GeoJSON: ^10^ closed contours become Polygon features, open contours LineString features, and cutting points Point features named by microsample identifier. Geometry is written in the pixel frame of the aligned overview image and carries the annotation properties expected by QuPath, ^11^ so contours and cutting positions can be imported directly onto the corresponding image, and are equally readable by Fiji/ImageJ, ^12^ napari, ^13^ or any GeoJSON-aware library. Because laser microdissection yields one bulk molecular profile per dissected region, these regions correspond to observations in the AnnData model ^14^ used by Scanpy, ^15^ and to columns of the expression matrix in Seurat. ^16^

### Operation

LMDmapper can be used as a packaged desktop application on Windows (10+), macOS (Monterey 12+), and Linux (Ubuntu 18.04+, Fedora 32+, Debian 10+) operating systems, which correspond to the nominal compatibility range of the embedded Electron runtime rather than an exhaustively tested matrix. Installers for each platform and architecture are built automatically for every release by the repository’s continuous-integration workflow.

**Figure 1.**
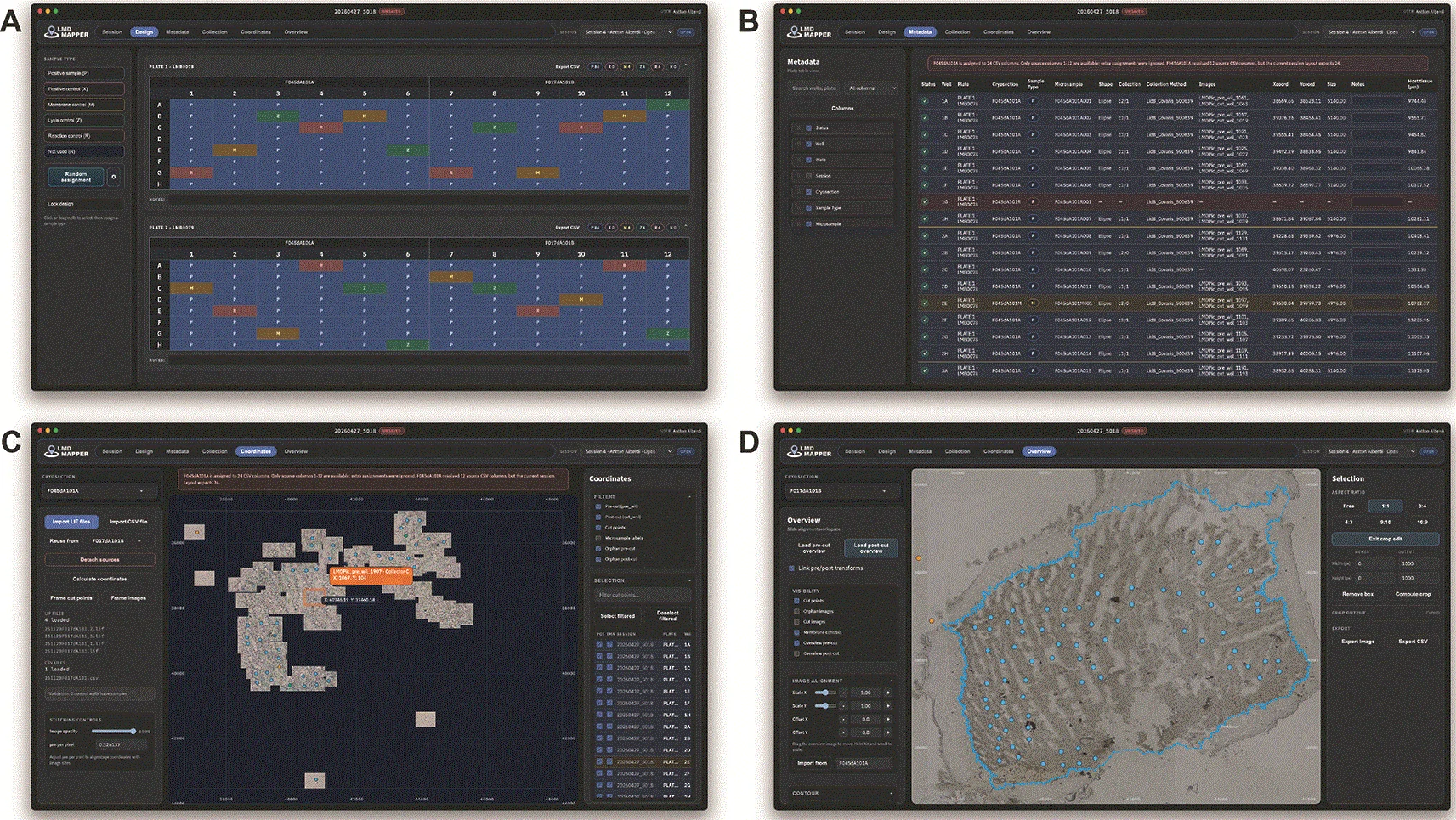
Screenshots of four workflow tabs. **A)** Design page showing the randomly assigned locations of different types of controls and regular samples across two plates. **B)** Metadata page containing sample details in tabular format. **C)** Coordinates page after loading the LIF and CSV files. Cutting points are indicated by blue markers, with the cursor position and details of the hovered image displayed in tooltips. A warning sign indicating unexpected sample information is shown above the canvas. **D)** Overview page showing cutting locations overlaid on a full-slide image. Blue dots represent regular samples, while orange dots represent membrane controls. A manually generated blue contour delineates the host tissue and is used to automatically calculate distances to the closest reference.

The software contains six workflow tabs that are used before (Project and Design), during (Collection) and after (Metadata, Coordinates, and Overview) the laser microdissection session ( Fig. 1). The expected user workflow is:

Before the LMD session:

1. **Start a session:** Define the session users to log changes.
2. **Configure the project**: Declare session name, cryosection or specimen identifiers, stage positions, number of plates, and plate-to-cryosection assignments. LMDmapper supports multiple sampling designs.
3. **Define the plate design**: For each of the microsample collecting plates, define the location of positive samples, controls, disabled wells, and microsample numbering ( Fig. 1A).

During the LMD session:

4. **Log microsample collection**: For each microsample cutting, declare the number of attempts for generating the sample and the number of microsamples visually checked in the collector. These data are logged in the collection tab, and displayed on the metadata table ( Fig. 1B).

After the LMD session:

5. **Import files**: Load Leica LIF files and laser microdissection CSV files for each cryosection to calculate laser microdissection locations and visualise them in a two-dimensional space ( Fig. 1C).
6. **Inspect the coordinate map**: Check image footprints, fix cut points, and detect missing or suspicious assignments.
7. **Enter collection metadata**: Enrich the metadata of each session with plate notes, collection method, date, time, temperature, humidity, and free-text notes.
8. **Align overview images**: Align pre-cut and post-cut overview images to the stage coordinate system, draw contours, compute crop cut positions.
9. **Export crops:** export the crop as a PNG with a JSON spatial reference, a CSV of crop and stage coordinates, and a GeoJSON of contours and cut points for third-party image analysis such as QuPath.
10. **Export metadata**: Choose visible and exported columns, and export a metadata CSV, including one contour-distance column per drawn contour ( Fig. 1D).
11. **Save the session**: As a ‘*.lmd’ file for a single session or combine multiple saved sessions into a ‘.mlmd’ workspace.

### Performance and scalability

Because laser microdissection sessions can combine several cryosections, multiple plates, and full-slide overview images, we characterised the file-handling performance of the tool on synthetic sessions of increasing size. The measurements exercise the same main-process functions the application calls over its inter-process interface: header parsing and element listing, on-demand thumbnail generation, full-resolution image loading, and session save and load ( Table 1).

**Table 1.** LMDmapper’s LIF file performance benchmarks. Details include session shape, image dimensions, access latency, and peak added memory.

Session shape	Images	Image size (px)	.lif size (MB)	XML header (KB)	List images (ms)	First image (ms)	Further images (ms)	Full-resolution image (ms)	Peak added memory (MB)
**Single cryosection**	24	1024 × 1024	72	18	2.1	44	6.9	0.8	100
**Two plates, one cryosection**	96	1024 × 1024	288	73	6.5	126	7.1	1.3	146
**Large field of view**	24	2048 × 2048	288	18	2.3	99	7.2	2.6	156
**Element-dense header**	500	256 × 256	94	381	24.8	43	3.0	0.2	98
**Full-slide overview**	2	12000 × 9000	618	2	0.8	179	6.1	75.0	245

Listing images took 0.8-25 ms across all cases, and was fastest for the 618 MB file because it contains only two image elements and therefore the smallest XML header. Displaying the first image of a file cost 43-179 ms, dominated by the one-time offset index; every further image was displayed in 3-7 ms, including a 12000 × 9000 pixel slide overview. Peak additional memory stayed below 160 MB while listing and browsing thumbnails. Loading a full-resolution element is the exception, costing time and memory of the order of the element itself: 75 ms and roughly 245 MB of transient memory for a 309 MB overview image. Session files remained small and fast: a session with 960 cut points occupied 0.5 MB and saved or loaded in under 2 ms, and a workspace merging six sessions with 2304 cut points in total occupied 1.7 MB and saved or loaded in about 4 ms.

Three implementation choices determine the positive scaling behaviour. First, listing the images in a LIF file reads only the ‘LMSDataContainerHeader’ XML section, streamed in 1 MB chunks, so listing cost follows the number of image elements rather than the size of the file. Second, image data is read on demand per element, and thumbnails are generated by strided row reads, so thumbnail memory depends on the requested thumbnail size and one source row rather than on the source image. Third, the first image displayed from a multi-element file triggers a single pass over the binary section that indexes every memory-block offset; every subsequent image is served from that cached index. One hard limit follows from the parser design: the XML header must be found within the first 128 MB of the file. Files whose header exceeds that limit are rejected with an explicit message rather than partially read. However, the header of a 500-element LIF file is 0.37 MB, so the limit corresponds to header sizes far beyond those produced by the sessions we have encountered.

## Use cases

### Curating a single laser microdissection session

A common LMDmapper use case is a session with one or two 96-well plates and one or more cryosections. The user first records the session name, cryosection identifiers, stage positions, and plate layout. Wells are classified as containing samples, controls, or not used. LMDmapper then generates microsample codes that remain tied to the plate and cryosection design.

**Figure 2.**
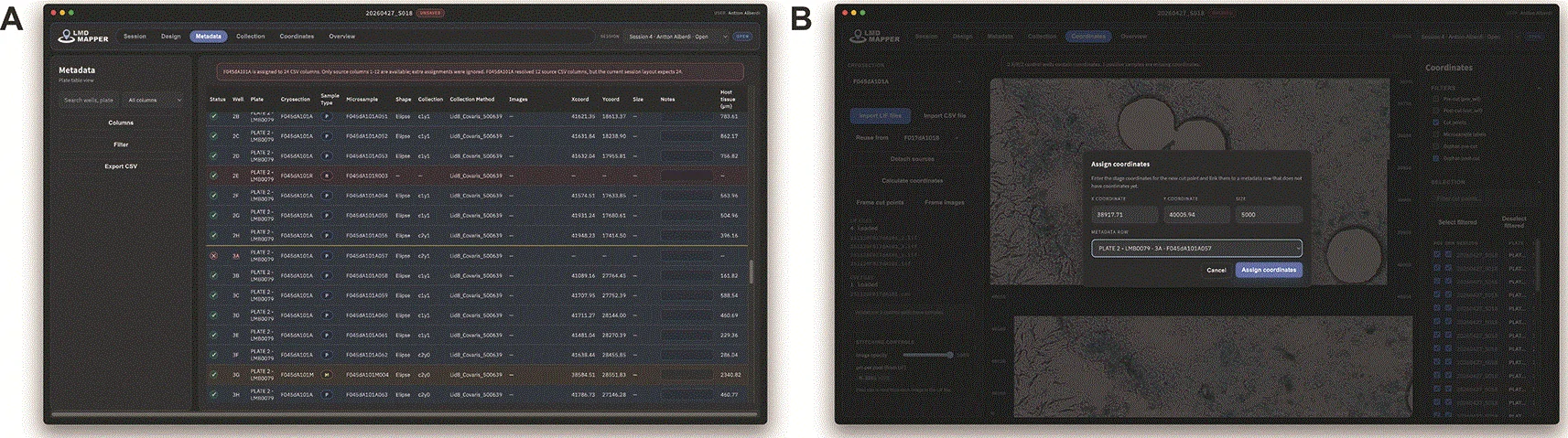
Screenshots representing the capabilities of LMDmapper. **A)** A metadata table that containing not only the cutting point coordinates (Xcoord, Ycoord) but also the measurements of each cutting point to a user-defined contour (Host tissue μm). Note that the differently coloured well 2E represents a reaction control with no coordinate information, while 3G indicates a membrane control. The well 3A is the only one with an error status, because LMDmapper was unable to find its corresponding image in the LIF file. **B)** A cutting points coordinates view over the canvas of spatially referenced microscopy images, where a window to manually assign a well membership to an orphan image is showcased.

The user imports the LIF files and CSV exports for each cryosection. LMDmapper parses the image metadata, reconstructs source columns from the CSV rows, routes the source columns onto the configured plate segments, and computes stage coordinates for wells with linked image names and pixel coordinates. The Metadata tab then provides one row per plate well, including plate, well, cryosection, sample type, microsample code, collection label, image labels, pixel coordinates, stage coordinates, size, status, notes, and optional distances to user-defined contours. Each row links the downstream microsample identifier to its tissue-derived coordinate evidence and collection context. For wells without valid coordinate evidence, LMDmapper marks the row as pending, warning, or bad depending on sample type and available information ( Fig. 2A).

### Resolving missing image links and orphan images

Laser microdissection exports may contain incomplete image labels or may be missing images. LMDmapper handles these cases by distinguishing linked image records from orphan images. Orphan images are image elements present in the ‘*.lif’ file but not assigned to a resolved metadata row.

In the Coordinates view, the user can filter orphan pre-cut and post-cut images, group them by collector row, inspect hover metadata, and assign an orphan image to a missing plate position. LMDmapper can also preserve original CSV pixel coordinates for a well even when its image link is removed, allowing the predicted cut marker to be displayed during reassignment. If no image evidence is available, the user can enter manual stage coordinates and link them to a metadata row. Manual coordinates and manual image assignments remain in warning status until explicitly confirmed, preserving an audit trail for downstream users.

The expected output is a corrected metadata table in which formerly missing or ambiguous wells have explicit manual-assignment status, image labels where available, resolved coordinates, and user notes describing the correction ( Fig. 2B).

### Exporting overview crops and spatial annotations

For reporting, quality control, or downstream spatial analysis, users may need an overview image crop that contains a subset of collected cut points. LMDmapper allows the user to import pre-cut and post-cut overview images, align them to the stage coordinate system, and show cut points, image footprints, orphan images, and control samples. The user can draw a selection rectangle, set an export size, compute which cut points fall within the crop, and export both the rendered crop image PNG and a CSV table of crop-local pixel coordinates. Users can also draw contours on the overview image, such as tissue boundaries or regions of interest. LMDmapper calculates the shortest distance from each cut point to each visible contour and exposes those distances as optional metadata columns.

## Discussion

LMDmapper addresses a practical reproducibility problem in laser microdissection workflows: Maintaining a transparent link between image evidence, spatial coordinates, plate positions, collection records, and downstream sample identifiers. By keeping the session state in structured local files and exporting plain CSV outputs, the tool is designed to fit into downstream omics and laboratory information-management workflows without requiring a server or proprietary database export.

LMDmapper is agnostic to the staining and to any other treatment the specimen underwent before laser microdissection, because it operates on the exported images and coordinates rather than on the tissue signal itself. Haematoxylin and eosin (H&E), immunohistochemistry (IHC), and immunofluorescence (IF) images can therefore all be used as reference images, whether they are read from LIF files or imported as overview layers. Alignment of images acquired from consecutive tissue sections, however, is not supported: overview alignment applies only scale and offset within a single section, and our own benchmarking showed that, at the spatial scale LMDmapper resolves, the tissue deformation between consecutive cryosections is too large to be corrected by such a transformation. Transferring regions of interest across serial sections therefore remains outside the current scope.

The application is most useful when a dissection workflow has enough complexity that manual spreadsheet curation becomes error-prone. Examples include split plates, multiple cryosections, positive and negative controls, shared CSV sources, re-cuts, missing image labels, manually corrected coordinates, and multiple saved sessions that need to be compared. The explicit warning states for inferred or manual assignments are intended to reduce hidden curation bias by making uncertain mappings visible at export time.

## Ethics and consent

Ethical approval and consent were not required.

## Data Availability

Source code available from:
-
github.com/anttonalberdi/lmdmapper
- Archived software available from: 10.5281/zenodo.20827562
- License: MIT License.
