# Supplementary material for: LMDmapper: an open-source desktop tool for spatial mapping of laser microdissection samples
Source: Open Res Eur. 2026 Sep 8;6:257. Originally published 2026 Jul 24. [Version 2] doi: 10.12688/openreseurope.24543.2 (PMC13586599; doi:10.12688/openreseurope.24543.2)
Supplement: Peer review history

# Reviewer report: Reviewer response for version 2

**DOI:** 10.21956/openreseurope.27160.r80661

**Published:** 2026-09-17

**Authors:** Pierluigi Di Chiaro

**Affiliations:** 1 Azienda Unità Sanitaria Locale-IRCCS di Reggio Emilia, Reggio Emilia, Italy

no further comments

Is the rationale for developing the new software tool clearly explained?

Yes

Is the description of the software tool technically sound?

Yes

Are sufficient details of the code, methods and analysis (if applicable) provided to allow replication of the software development and its use by others?

Partly

Is sufficient information provided to allow interpretation of the expected output datasets and any results generated using the tool?

Partly

Reviewer Expertise:

expert in spatial transcriptomics and data analysis including  LMD applications

I confirm that I have read this submission and believe that I have an appropriate level of expertise to confirm that it is of an acceptable scientific standard.

# Reviewer report: Reviewer response for version 1

**DOI:** 10.21956/openreseurope.26589.r78198

**Published:** 2026-08-14

**Authors:** Yuliaxis Ramayo

**Affiliations:** 1 Institute for Research and Technology in Food and Agriculture (IRTA), Torre Marimon, Caldes de Montbui, Spain

Here, the authors present *LMDmapper*, an open-source Electron/React desktop application designed to reconcile Leica LIF stage coordinates with laser microdissection CSV pixel-coordinate exports, producing a unified metadata table that links microsample identifiers to plate positions, image evidence, and Cartesian coordinates.  The tool addresses a genuine and, to the best of my knowledge, previously unmet need.  I just have few minor suggestions for the authors

**Minor Comments:**

1. *No performance or scalability data.* Sessions with multiple plates, multiple cryosections, and full-slide overview images could plausibly involve large LIF files and many hundreds of cutting points. A brief note on tested dataset sizes, memory footprint, or loading times (even approximate) would help prospective users anticipate whether the tool will hold up on their own session sizes.
2. The "Operation" section lists supported OS versions (Windows 10+, macOS Monterey 12+, Ubuntu 18.04+, Fedora 32+, Debian 10+) but does not state whether these were all actually tested, or whether this reflects Electron's nominal compatibility range; worth clarifying which.
3. Figure 1's caption is informative, but the main text never explicitly points the reader to panel (b) when describing metadata entry (step 7) , a small cross-referencing gap.

Is the rationale for developing the new software tool clearly explained?

Yes

Is the description of the software tool technically sound?

Yes

Are sufficient details of the code, methods and analysis (if applicable) provided to allow replication of the software development and its use by others?

Yes

Is sufficient information provided to allow interpretation of the expected output datasets and any results generated using the tool?

Yes

Reviewer Expertise:

Computational biology, hologenomics

I confirm that I have read this submission and believe that I have an appropriate level of expertise to confirm that it is of an acceptable scientific standard.

# Response

**Published:** 2026-08-28

**Authors:** Antton Alberdi

We appreciate the positive comment of the reviewer. Minor Comments: No performance or scalability data. Sessions with multiple plates, multiple cryosections, and full-slide overview images could plausibly involve large LIF files and many hundreds of cutting points. A brief note on tested dataset sizes, memory footprint, or loading times (even approximate) would help prospective users anticipate whether the tool will hold up on their own session sizes. >> Following this request, we have added a performance characterisation in the manuscript  (Methods, "Performance and scalability", Table 2). Sessions were benchmarked at 24–500 images per .lif file, file sizes of 72–618 MB, individual images up to 12000 × 9000 pixels, and sessions of up to 960 cut points, with merged workspaces of 2304 cut points. Listing the images in a file takes 0.8–25 ms and depends on the number of image elements rather than file size; the first image displayed from a file costs 43–179 ms because of a one-time memory-block index pass, and every further image 3–7 ms irrespective of its dimensions. Peak additional memory stays below 160 MB while browsing, rising to roughly the size of the image only when a full-resolution element is displayed. Session files remain under 2 MB and save or load in a few milliseconds.  The benchmark is now included in the repository as npm run benchmark, and the full output, method, and known limits (including the 128 MB .lif header ceiling) are documented in docs/benchmark.md, so the measurements can be reproduced on other hardware.

 The "Operation" section lists supported OS versions (Windows 10+, macOS Monterey 12+, Ubuntu 18.04+, Fedora 32+, Debian 10+) but does not state whether these were all actually tested, or whether this reflects Electron's nominal compatibility range; worth clarifying which. >> The listed versions are the nominal compatibility range of the Electron runtime that LMDmapper embeds (Electron 40), not a matrix of operating systems on which we have run the application. Specifically, the macOS binary produced by this Electron version declares a minimum system version of 12.0, and Windows 10 or later and glibc 2.28-based distributions (Ubuntu 18.04, Debian 10, Fedora 32 and later) are the platforms declared as supported by the Electron project. Installers for all distributed platform and architecture combinations are built automatically for every release by the continuous-integration workflow included in the repository, using the macOS (Apple silicon and Intel), Windows, and Ubuntu 24.04 (x64 and arm64) runner images of GitHub Actions; the same workflow lints and builds the source tree on Linux for every change. Figure 1's caption is informative, but the main text never explicitly points the reader to panel (b) when describing metadata entry (step 7) , a small cross-referencing gap. >> We have now added a reference to 1b into the manuscript text.

# Reviewer report: Reviewer response for version 1

**DOI:** 10.21956/openreseurope.26589.r78196

**Published:** 2026-08-12

**Authors:** Pierluigi Di Chiaro

**Affiliations:** 1 Azienda Unità Sanitaria Locale-IRCCS di Reggio Emilia, Reggio Emilia, Italy

The authors developed LMDmapper, an open-source desktop application for designing, tracking, and documenting micron-scale spatial samples and associated metadata generated during laser microdissection sessions. This software addresses an important gap in the LMD field by providing a structured system for organizing images, sample information, and experimental metadata after microdissection. However, several aspects of the manuscript are not yet sufficiently clear for potential users. In particular, some software functions and workflows require a more detailed visual explanation, and the advantages, limitations, and possible downstream applications of the exported outputs should be discussed more clearly.

**Improve the quality and readability of Figure 1.**

 The resolution of the screenshots should be increased, as several elements are currently difficult to read. Rectangles, arrows, numbered labels, or short annotations should be added to better indicate the specific function illustrated in each panel. In particular, panel D should more clearly show how to align overview images, draw contours, compute crop-cut positions, and export overview crops as PNG files together with the corresponding CSV files. Additional screenshots or intermediate panels should be included, if necessary, to make these steps easier to understand.

**Add a figure illustrating the main use cases of LMDmapper.**

 Similarly, a dedicated figure should be added to the Use Cases section. This would help clarify how the software can be used to resolve missing image links and orphan images, as well as to export overview crops and spatial annotations.

**Briefly expand the Discussion on the advantages and limitations of LMDmapper in the era of spatial biology.**

 The authors should more clearly discuss the advantages and limitations of their tool. For example, they should clarify whether additional H&E, IHC, or IF images acquired from consecutive tissue sections can be imported and aligned with the image used for LMD. Another important point is whether the exported images, contours, and spatial coordinates of the microdissected regions can be used in third-party software, such as QuPath for image analysis, or integrated with molecular outputs, such as transcriptomic data, using tools such as Seurat or Scanpy.

Is the rationale for developing the new software tool clearly explained?

Yes

Is the description of the software tool technically sound?

Yes

Are sufficient details of the code, methods and analysis (if applicable) provided to allow replication of the software development and its use by others?

Partly

Is sufficient information provided to allow interpretation of the expected output datasets and any results generated using the tool?

Partly

Reviewer Expertise:

expert in spatial transcriptomics and data analysis including  LMD applications

I confirm that I have read this submission and believe that I have an appropriate level of expertise to confirm that it is of an acceptable scientific standard, however I have significant reservations, as outlined above.

# Response

**Published:** 2026-08-28

**Authors:** Antton Alberdi

We appreciate the timely review of our manuscript. We address the reviewer’s comments in the following.

 Improve the quality and readability of Figure 1. >> We have uploaded a new version of the figure in a different format. The resolution of the screenshots should be increased, as several elements are currently difficult to read. Rectangles, arrows, numbered labels, or short annotations should be added to better indicate the specific function illustrated in each panel. In particular, panel D should more clearly show how to align overview images, draw contours, compute crop-cut positions, and export overview crops as PNG files together with the corresponding CSV files. Additional screenshots or intermediate panels should be included, if necessary, to make these steps easier to understand. >> Following this request, we have increased the resolution of the images. However, instead of adding new images in this initial figure, we have now included a figure containing two more screenshots to better explain the functionality of LMDmapper. These figures incorporate the details requested by the reviewer in this comment. Add a figure illustrating the main use cases of LMDmapper. Similarly, a dedicated figure should be added to the Use Cases section. This would help clarify how the software can be used to resolve missing image links and orphan images, as well as to export overview crops and spatial annotations. >> See response above. Briefly expand the Discussion on the advantages and limitations of LMDmapper in the era of spatial biology. The authors should more clearly discuss the advantages and limitations of their tool. For example, they should clarify whether additional H&E, IHC, or IF images acquired from consecutive tissue sections can be imported and aligned with the image used for LMD. >> We have expanded the Discussion to address this. Another important point is whether the exported images, contours, and spatial coordinates of the microdissected regions can be used in third-party software, such as QuPath for image analysis, or integrated with molecular outputs, such as transcriptomic data, using tools such as Seurat or Scanpy. >> Following this request, we have released a new version, 1.5.0, which includes the possibility to export contours as an RFC 7946 GeoJSON file. Geometry is written in the pixel frame of the aligned overview image and carries QuPath's annotation properties, and is equally readable by ImageJ/Fiji, napari, or any GeoJSON-aware library. This is now mentioned in the manuscript as well.
